# Sequential Changes in Brain Glutamate and Adenosine A1 Receptors May Explain Severity of Adolescent Alcohol Withdrawal after Consumption of High Levels of Alcohol

**DOI:** 10.1155/2019/5950818

**Published:** 2019-06-02

**Authors:** Patrycja Bolewska, Bryan I. Martin, Krystal A. Orlando, Dennis E. Rhoads

**Affiliations:** Department of Biology, Monmouth University, W. Long Branch, NJ 07764, USA

## Abstract

There is an excellent correlation between the age when alcohol consumption begins and the likelihood of lifelong problems with alcohol abuse. Alcohol use often begins in adolescence, a time marked by brain development and maturation of numerous brain systems. Rats are an important model, wherein the emergence of alcohol withdrawal symptoms serves as a gauge of dependency following chronic alcohol consumption. Previous work has shown that adolescent Long-Evans rats consume high levels of alcohol and develop a severe alcohol withdrawal syndrome when fed alcohol as part of a liquid diet. Acutely, alcohol inhibits two important excitatory receptors for glutamate (NMDA and AMPA) and may further decrease glutamate activity through modulatory adenosine receptors. The present study focuses on potential adaptive changes in expression of these receptors that may create a receptor imbalance during chronic alcohol consumption and lead to severe overexcitation of the adolescent brain during alcohol withdrawal. Levels of brain expression of NMDA, AMPA, and adenosine A1 and A2a receptors were determined by Western blotting after adolescent rats consumed an alcohol-containing liquid diet for 4, 11, or 18 days. Severity of alcohol withdrawal was also assessed at these time points. Levels increased for both AMPA and NMDA receptors, significant and approaching maximal by day 11. In contrast, A1 receptor density showed a slow decline reaching significance at 18 days. There were no changes in expression of adenosine A2a receptor. The most severe withdrawal symptoms appear to coincide with the later downregulation of adenosine A1 receptors coming on top of maximal upregulation of excitatory AMPA and NMDA glutamate receptors. Thus, loss of adenosine “brakes” on glutamate excitation may punctuate receptor imbalance in alcohol-consuming adolescents by allowing the upregulation of the excitatory receptors to have full impact during early alcohol withdrawal.

## 1. Introduction

Adaptive changes during chronic alcohol consumption lead to a hyperexcitable state most closely associated with an overactive glutamatergic system (for recent reviews, see [1–3]). Particularly important is widespread upregulation of the N-methyl-D-aspartate (NMDA) subtype of glutamate receptor. Upregulation of NMDA receptors occurs in the cerebral cortex and a number of subcortical regions. Thus, the adaptive increase in NMDA receptors has been implicated in a number of symptoms associated with alcohol withdrawal and the resulting negative affective state [2]. As withdrawal symptoms appear, they can lead the individual to return to drinking alcohol, essentially self-medicating to alleviate these adverse symptoms [4–6]. This may be especially important during the periods of acute withdrawal and early abstinence [7].

Adolescence is a time when many individuals have their first exposure to alcohol and represents a period of brain development that provides a unique substrate behaviorally and biochemically for drug exposure (reviewed in [8–13]). Differences in the adolescent brain may underlie the tendency toward binge drinking in adolescence. Again, NMDA receptors appear to be important. For example, alcohol more potently inhibits NMDA receptor activity acutely in the adolescent brain [14].

When given alcohol as part of a liquid diet, adolescent rats consume high levels of alcohol, achieve blood alcohol concentrations well above the threshold for binge drinking among human adolescents, and develop a severe alcohol withdrawal syndrome [15–19]. The latter is marked by a high percentage of convulsive episodes during the withdrawal period. Applied through liquid diets, ethanol consumption and severity of the resulting alcohol withdrawal syndrome each decreased as the rats aged through and beyond the periadolescent period [15]. Liver alcohol metabolism and ethanol elimination rates were as high or higher in adolescent rats compared to adults [15]. We have shown that the severity of withdrawal symptoms for adolescent rats progresses from relatively mild symptoms after consuming the alcohol-containing diet for 4-10 days to a high percentage of convulsions or apparent seizures if alcohol consumption continues beyond two weeks [18]. Thus, this provides a good model system to explore biochemical changes in the adolescent brain during chronic consumption of alcohol. In addition, liquid diets can be exploited to study coadministration of other drugs with alcohol. We have used this system to show that administration of alcohol with either caffeine [18] or amphetamine [19] decreased the severity of subsequent alcohol withdrawal. This adds to the importance of understanding biochemical changes occurring during chronic alcohol consumption.

In the present study, we have focused on three time points (short, intermediate, and prolonged) for duration of alcohol consumption and thus progression of alcohol withdrawal severity to determine if alterations in NMDA receptors occur during adolescent alcohol consumption under these conditions. We also examined expression of the *α*-amino-3-hydroxy-5-methyl-4-isoxazolepropionic acid (AMPA) subtype of glutamate receptor. While they are less studied than NMDA with respect to ethanol, AMPA receptors have been shown to be inhibited by acute ethanol [20, 21] and in some cases to be upregulated by chronic ethanol exposure [22–24]. The glutamate system is also subject to negative modulation by the adenosine system and adenosine is elevated by alcohol consumption (reviewed in [25]). This may be particularly relevant in this system because we have shown that caffeine, an antagonist of adenosine receptors, alters withdrawal severity when administered before and during chronic alcohol consumption [18]. Therefore, we have also determined the effect of chronic alcohol consumption on the A1 and A2a subtypes of adenosine receptor.

## 2. Materials and Methods

### 2.1. Animals and Ethanol Feeding

Juvenile male LE rats were obtained from Charles River Laboratories (Raleigh, NC) and used after a minimum of 3 days of acclimation to our animal facility. Rats were maintained in a controlled temperature and humidity environment with a light cycle from 07:00 to 19:00. In studies of chronic alcohol consumption, rats were housed individually and fed a preformulated liquid diet [26] (LD'82 Liquidiet, Bioserv Inc., Frenchtown, NJ). At a final concentration of 3.5% (w/v), ethanol was administered in the liquid diet beginning at postnatal day 28 (P28) and continuing up to 18 days as described previously [15, 18, 19]. This coincides with a starting point in early adolescence for rats [8]. Rats had unlimited access to the ethanol-containing diet and the amount of diet consumed was recorded daily for each rat. Age-matched controls were pair-fed an ethanol-free liquid diet or given free access to rat chow and water. All protocols involving rats were reviewed and approved by the Institutional Animal Care and Use Committee of Monmouth University as prescribed in the Public Health Service Guide for Care and Use of Laboratory Animals.

### 2.2. Severity of Withdrawal Symptoms

Based on previous work [15, 18, 19], rats were evaluated for severity of ethanol withdrawal beginning at 6 hours after being switched from the ethanol-containing diet to a control formula (no ethanol) liquid diet (Bioserv Inc.) or simply to regular rat chow and tap water to affect ethanol withdrawal. Based on the characterization of the alcohol withdrawal syndrome in adult rats by Majchrowicz [27], the scoring system of Penland et al. [28] was used to assess the severity of withdrawal symptoms as described previously [18, 19]. This is a 4-point scale, including scores of 1.0 for general hyperactivity, 2.0 for caudal region tremors, 3.0 for head tremors, 3.2 for induced running episodes, and 3.8 for convulsions during withdrawal. In general, the strongest withdrawal symptoms seen in the present study were convulsions and apparent seizures following a quickly escalating progression of hyperreflexia and running episodes.

### 2.3. Fractionation and Western Blotting

Ethanol-fed or pair-fed control rats were euthanized by rapid decapitation after 4, 11, or 18 days of liquid diet consumption. Rats were not withdrawn from alcohol-containing diets before euthanizing them. The forebrain was dissected on ice and fractionated as described previously [29]. Proteins were solubilized by heating at 95C for 5 min in Laemmli sample buffer (Bio-Rad, Hercules, CA) and separated by polyacrylamide gel electrophoresis (10% Bio-Rad ready gel) under constant voltage (200 V). For each membrane fraction, 10-30 *μ*g of protein was loaded per lane based on preliminary analyses showing this was within the linear range for detection. Proteins were transferred to PVDF membranes using a Mini Trans-Blot cell (Bio-Rad). Western blotting proceeded using the SNAP i.d. Protein Detection System (Millipore Sigma, Burlington MA) following the manufacturer's protocol. The blots were blocked with nonfat dry milk (Bio-Rad), washes were with Tris-buffered saline containing 1% (v/v) Tween, primary antibodies were used at 1:500 dilution, and goat anti-rabbit peroxidase-conjugated secondary antibody (MilliporeSigma) was used at 1:2000 dilution. Rabbit monoclonal antibodies were obtained for NMDA receptor NR1 (clone 1.17.2.6; MilliporeSigma), the AMPA receptor GluR1 (clone C3T; MilliporeSigma), and actin (clone D6A8; Cell Signaling Technology, Danvers, MA). Rabbit polyclonal antibodies (MilliporeSigma) were obtained for adenosine A1 receptor (AB1587p) and adenosine A2a receptor (AB1559). For NMDA and AMPA receptor antibodies, blots were simultaneously probed for actin as an internal loading control. For adenosine A1 and A2a blots were stripped (ReBlot Plus, MilliporeSigma) and reprobed for actin. In all cases, detection was by chemiluminescent product formation (SuperSignal West Pico Chemiluminescent Substrate, Thermo Scientific) which was quantified using the ChemiDoc XRS+ system with Image Lab software (Bio-Rad). Density of proteins detected in each treatment group was expressed relative to controls set at 1.

### 2.4. Statistical Analysis

All values presented are means ± standard errors. All statistical analyses were performed using ProStat software (Poly Software International, Pearl River, NY) or GraphPad InStat (San Diego, CA). Differences in protein expression between pair-fed control and ethanol-fed rats were evaluated at the 18th day time point by 2-tailed Student's* t*-test. For duration of alcohol consumption, differences in expression were evaluated by two-factor ANOVA (2 treatment groups x 3 time points). Where a significant main effect of treatment was found, individual comparisons were made using Tukey's post test. In all cases, significance was set at* p* < 0.05.

## 3. Results

**Figure 1:**
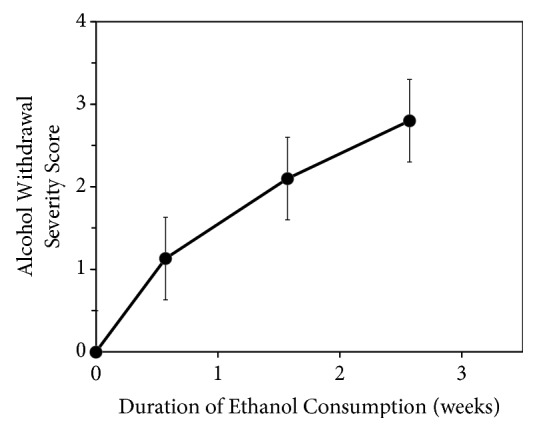
*Alcohol withdrawal severity measured at three time points for duration of alcohol consumption.* A 4-point scale was used to assess the severity of alcohol withdrawal symptoms after 4, 11, and 18 days of alcohol consumption beginning at P25. Results are mean ± standard error of 12 measures for each time point.

On average, the rats consumed 15.9 ± 0.4 g ethanol per day per kg body weight over the course of the administration period. Withdrawal severity was low (1.1) after 4 days and then increased to 2.1 after 11 days and to 2.8 after 18 days of alcohol consumption (Figure 1). The high withdrawal severity score after 18 days of alcohol consumption comes from the high percentage of rats showing convulsions or apparent seizures following a quickly escalating progression of hyperreflexia and running episodes.

**Figure 2:**
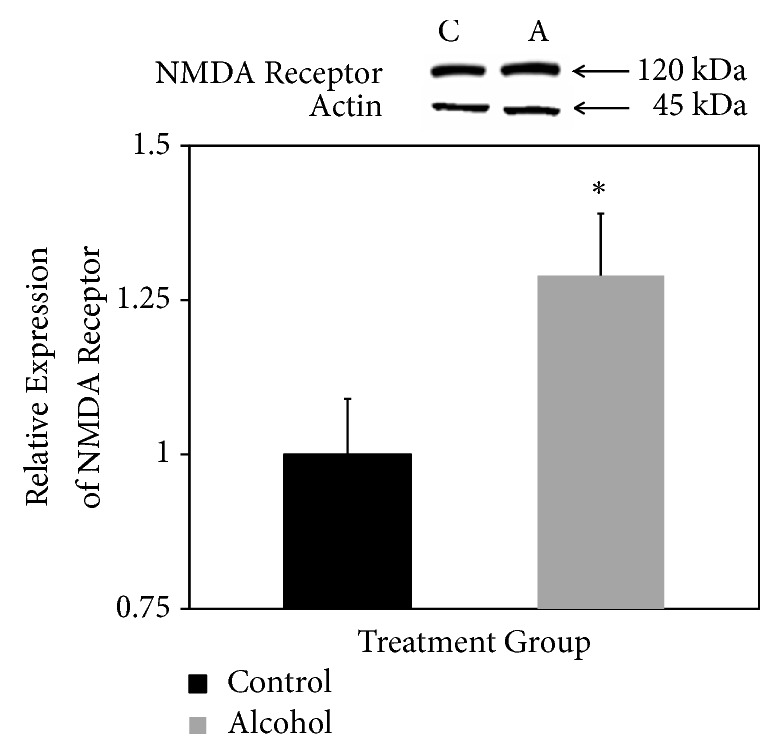
*Relative expression of NMDA receptor.* Western blotting was used to detect the 120 kDa NMDA receptor in forebrain fractions of rats fed liquid diets with (A, alcohol) and without (C, control) ethanol. Results are expressed relative to expression of actin as an internal loading control with control levels set at 1.0. Results are means ± standard error of 6 determinations. *∗ p* < 0.05.

NMDA receptor was detected at the expected mass of 120 kDa after SDS-PAGE and Western blotting (Figure 2, top panel). Bands appeared darker in samples derived from rats consuming alcohol for 18 days (Figure 2, top panel). Normalized to actin expression, NMDA receptor expression was confirmed to be significantly higher in rats that consumed alcohol for 18 days (Figure 2, bottom panel).

**Figure 3:**
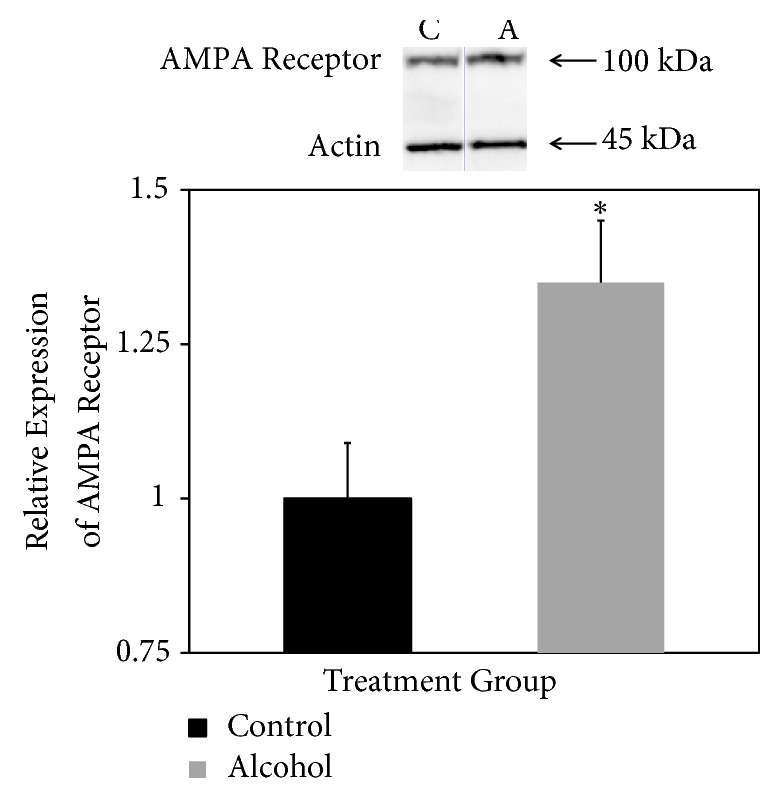
*Relative expression of AMPA receptor.* Western blotting was used to detect the 100 kDa AMPA receptor in forebrain fractions of rats fed liquid diets with (A, alcohol) and without (C, control) ethanol. Results are expressed relative to expression of actin as an internal loading control with control levels set at 1.0. Results are means ± standard error of 4 determinations. *∗ p* < 0.05.

AMPA receptor was detected at the expected mass of 100 kDa after SDS-PAGE and Western blotting (Figure 3, top panel). As with NMDA, bands appeared darker in samples derived from rats consuming alcohol (Figure 3, top panel). Normalized to actin expression, AMPA receptor expression was confirmed to be significantly higher in rats that consumed alcohol for 18 days (Figure 3, bottom panel).

**Figure 4:**
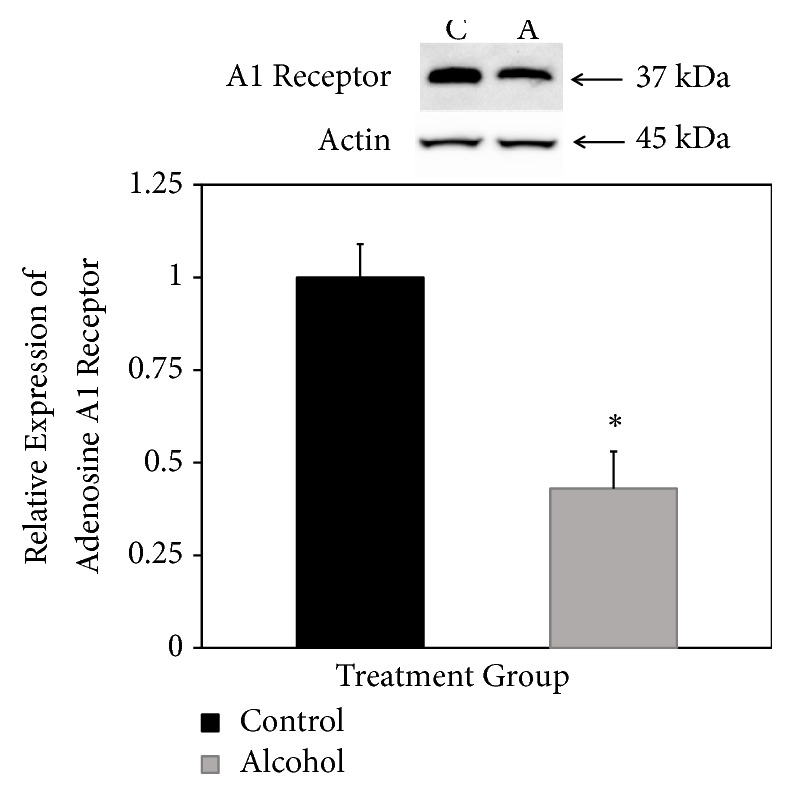
*Relative expression of adenosine A1 receptor.* Western blotting was used to detect the 37 kDa A1 receptor in forebrain fractions of rats fed liquid diets with (A, alcohol) and without (C, control) ethanol. Results are expressed relative to expression of actin as an internal loading control with control levels set at 1.0. Results are means ± standard error of 6 determinations. *∗ p* < 0.05.

Adenosine A1 receptor was detected at the expected mass of 37 kDa after SDS-PAGE and Western blotting (Figure 4, top panel). In this case, bands appeared lighter in samples derived from rats consuming alcohol (Figure 4, top panel). Normalized to actin expression, A1 receptor expression was confirmed to be significantly lower in rats that consumed alcohol for 18 days (Figure 4, bottom panel).

**Figure 5:**
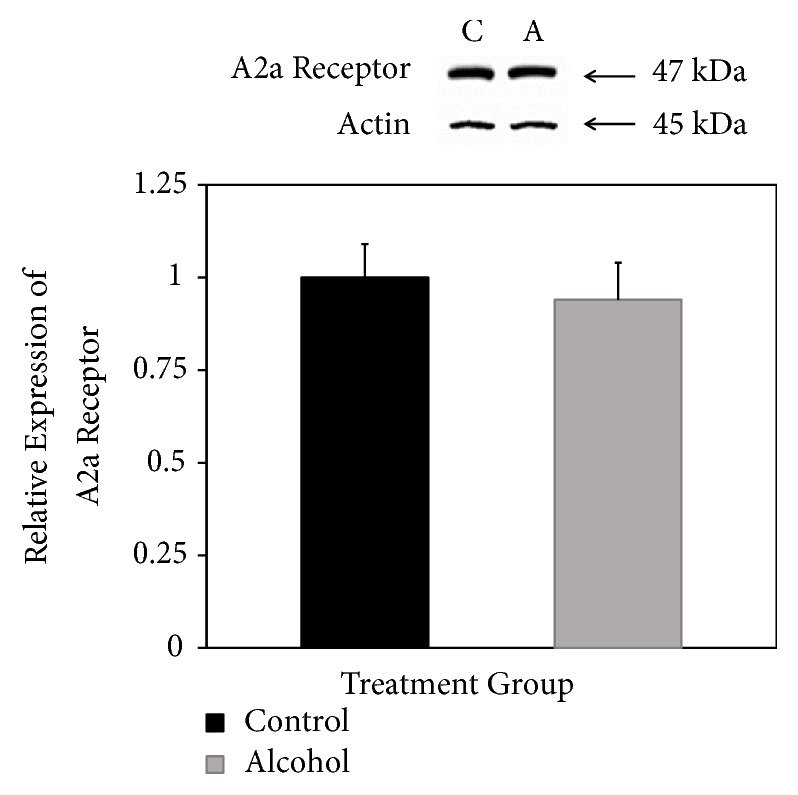
*Relative expression of adenosine A2a receptor.* Western blotting was used to detect the 47 kDa A2a receptor in forebrain fractions of rats fed liquid diets with (A, alcohol) and without (C, control) ethanol. Results are expressed relative to expression of actin as an internal loading control with control levels set at 1.0. Results are means ± standard error of 4 determinations. *∗ p* < 0.05.

Adenosine A2a receptor was detected at the expected mass of 47 kDa after SDS-PAGE and Western blotting (Figure 5, top panel). In this case, there was no obvious difference when samples were derived from rats consuming alcohol (Figure 5, top panel). Normalized to actin expression, A2a receptor expression was shown not to be significantly different in rats that consumed alcohol for 18 days (Figure 5, bottom panel).

**Figure 6:**
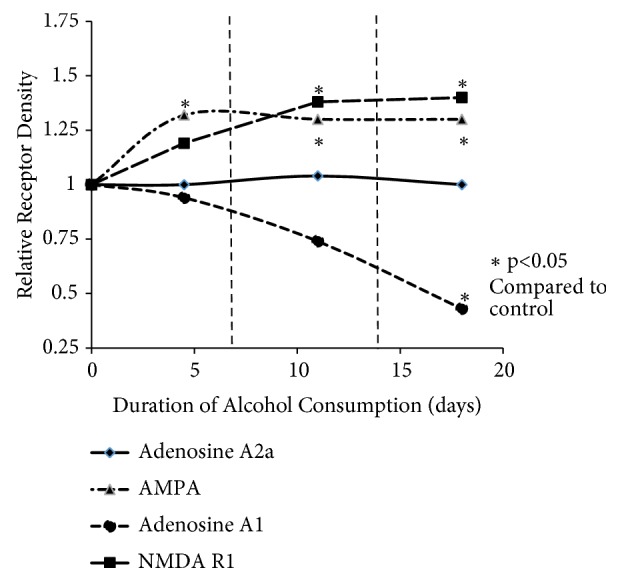
*Relative expression of receptors over 3 time points for duration of alcohol consumption.* Western blotting was used to detect the NMDA, AMPA, adenosine A1, and adenosine A2 receptors in forebrain fractions of rats fed liquid diets containing ethanol for periods of 4, 11, and 18 days. Results are expressed relative to receptor expression for age-matched control rats consuming the ethanol-free diet for the same period. Results are means ± standard error of 3 determinations. *∗ p* < 0.05.

Receptor expression was measured after 4, 11, and 18 days of alcohol consumption and evaluated by two-factor ANOVA (treatment group x duration of diet consumption) (Figure 6). For NMDA receptor expression, there was a significant main effect of ethanol consumption [F (1,17) = 38.934, p<0.01] but no significant main effect of duration and no significant interaction effect between treatment and duration (p=0.23). Posttest analyses showed that NMDA expression was significantly higher after 11 (p<0.05) and 18 (p<0.01) days of alcohol consumption. There was a trend toward increased expression at 4 days but this difference was not significant (p=0.34). For AMPA receptor expression, there was a significant main effect of ethanol consumption [F (1,17) = 36.853, p<0.01] but again there was no significant main effect of duration and no significant interaction effect between treatment and duration (p=0.98). Posttest analyses showed that AMPA expression was significantly higher after 4 (p=0.03), 11 (p<0.05), and 18 (p<0.05) days of alcohol consumption. Adenosine A1 receptor expression showed a significant main effect of ethanol consumption [F (1,17) = 17.934, p<0.01], a significant main effect of duration [F (2,17) = 4.485, p<0.05], and a significant interaction effect between ethanol consumption and duration [F (2,17) = 4.485, p<0.05]. Posttest analyses showed that decreased expression of adenosine A1 receptor was significant only after 18 days of ethanol consumption (p<0.05). There was a trend toward decreased expression of A1 receptor after 4 and 11 days of alcohol consumption but these differences were not significant. With expression of adenosine A2a receptor, there was no significant main effect of either ethanol consumption (p=0.82) or duration (p=0.95) and no significant interaction effect (p=0.95).

## 4. Discussion

Alcohol-containing liquid diets provided an approach leading to high levels of alcohol consumption by adolescent rats and progressively severe withdrawal symptoms over time. Alcohol consumption and severity of withdrawal reported here are consistent with our previous reports using Long-Evans adolescents and liquid diets as the source of alcohol [15, 17–19]. It is important to note that the interruptions to the alcohol administration schedule in this study to allow for withdrawal measures at 4 and 11 days had no significant impact on the final withdrawal severity compared to our previous reports with continuous uninterrupted alcohol administration for the entire period. Such interruptions (multiple withdrawals) in chronic intermittent alcohol exposure can lead to more severe withdrawal symptoms in rats and mice ([4, 7, 30]. The consumption of the liquid diet coupled with high rates of adolescent alcohol metabolism may already lead to some kind of kindling effect as withdrawal severity (and apparent seizure frequency) is high for these adolescent Long-Evans rats even without multiple withdrawals [15, 18, 19].

Expression of both ionotropic glutamate receptors, AMPA and NMDA, increased during the period of alcohol administration. We chose to use a simple forebrain preparation in this study because of the widespread distribution of AMPA and NMDA receptors [1, 2]. However, preliminary results have confirmed our major findings with similar preparations from adolescent frontal cortical areas known to be important targets for alcohol and other drugs [11]. The increase in levels of NMDA receptor is consistent with reports from numerous studies including multiple systems and modes of delivering alcohol (reviewed in [1–3]). Acutely, alcohol inhibits the NMDA receptor and upregulation of NMDA receptor expression would appear to be compensatory. Changes in AMPA receptor levels have been seen in some but not all studies (for recent review, see [3]). There is evidence for inhibition of AMPA receptor activity acutely by ethanol and so again upregulation could be compensatory. However, increased AMPA receptor expression is also associated with NMDA receptor activity and so could be secondary to the changes occurring in NMDA receptor [31]. The limited time course conducted in the present study does not allow a firm conclusion regarding whether one occurred first. There was a trend toward increases in both receptors within the first week (at 4-5 days); however, only the increase in AMPA was statistically significant at that time point. Increases in both AMPA and NMDA reached significance in the second week of alcohol consumption (at 11 days). Although only 3 time points were tested, it appeared that glutamate receptor expression was reaching a maximum by 11 days of alcohol consumption. This is of interest because withdrawal severity appeared to continue to increase to 18 days.

The most severe withdrawal symptoms correspond to the point where expression of adenosine A1 receptor reaches a significantly low level. There was no effect of ethanol on adenosine A2a receptor expression. Although A2a receptors may become desensitized with prolonged ethanol exposure [32], the current finding that A2a receptor levels remain unchanged following chronic alcohol exposure is in agreement with a number of other studies (reviewed in [25]). Likewise, there is general agreement that A1 receptor levels change temporarily after chronic alcohol exposure; however, the exact change appears sensitive to the conditions [25]. In the earliest study of this type, Dar and coworkers [33] found decreases in Bmax for A1 receptor ligand binding in homogenates of mouse brain following long-term ethanol exposure. In contrast, several studies have reported no change in A1 receptor levels immediately upon withdrawal but significant increases in A1 levels as withdrawal proceeds [30, 34, 35]. Changes in expression of adenosine transporter have been found in the absence of changes in A1 or A2a receptor levels [36]. In comparing our results to these previous studies, we also saw no statistically significant change in A1 levels until the 3rd week (18th day time point). The large decrease in A1 receptor expression seen in the present study would thus appear to result from three interrelated factors: (1) the use of adolescent rats as the model, (2) high levels of alcohol consumption through liquid diets and resulting high blood alcohol levels, and (3) perhaps, most importantly, the duration of alcohol exposure being beyond two weeks to reach statistical significance.

The consequences of reduced A1 levels can be seen in the effects of A1 antagonists during alcohol withdrawal. Reducing A1 activity with a receptor antagonist significantly increased alcohol withdrawal severity [37]. Further support for the view that adenosine receptors modulate withdrawal severity comes from studies showing that withdrawal severity is decreased by A1 agonists [36, 38] and also by chronic exposure to caffeine [18] which may trigger adaptive changes in A1 levels [39]. A clear link to glutamate and NMDA receptor activity is seen in studies of female hippocampal cultures where toxicity produced by an A1 receptor antagonist or caffeine during ethanol withdrawal was abolished by an NMDA receptor antagonist [40, 41]. Our direct demonstration of effects of ethanol on expression of A1 receptors complements these indirect approaches to characterizing the importance of A1 receptors through the effects agonists and antagonists. The reduction in the modulatory A1 receptors may contribute along with alteration in GABA reception [42] to overall decreased inhibition during chronic alcohol consumption.

## 5. Conclusions

We conclude that decrease in A1 receptor levels coupled with increases in excitatory glutamate receptors may underlie the severe alcohol withdrawal syndrome seen in adolescent LE rats. Loss of adenosine “brakes” on glutamate excitation may complete the receptor imbalance in alcohol-consuming adolescents and allows the upregulation of the excitatory receptors to have their full impact during early alcohol withdrawal.

## Data Availability

The raw data used to support the findings of this study are available from the corresponding author upon request.
